# Epigenetic Factors in Late-Onset Alzheimer’s Disease: *MTHFR* and *CTH* Gene Polymorphisms, Metabolic Transsulfuration and Methylation Pathways, and B Vitamins

**DOI:** 10.3390/ijms20020319

**Published:** 2019-01-14

**Authors:** Gustavo C. Román, Oscar Mancera-Páez, Camilo Bernal

**Affiliations:** 1 Department of Neurology, Methodist Neurological Institute, Institute for Academic Medicine Houston Methodist Research Institute, Houston Methodist Hospital, Houston, TX 77030, USA; 2 Weill Cornell Medical College, Department of Neurology, Cornell University, New York, NY 10065, USA; 3 Universidad Nacional de Colombia, Hospital Universitario Nacional, Faculty of Medicine, Department of Neurology, Bogotá ZC 57, Colombia; ogmancerap@unal.edu.co (O.M.-P.); camilobernalmd@hotmail.com (C.B.); 4 David Cabello International Alzheimer Disease Scholarship Fund, Houston Methodist Hospital, Houston, TX77030, USA

**Keywords:** Alzheimer’s disease, cystathionine-γ-lyase *CTH* gene, DNA methylation, epigenetics, epigenome-wide association study, methylome, methylenetetrahydrofolate reductase *MTHFR* gene, nutrition, S-adenosylmethionine, vitamin B complex

## Abstract

DNA methylation and other epigenetic factors are important in the pathogenesis of late-onset Alzheimer’s disease (LOAD). Methylenetetrahydrofolate reductase (*MTHFR*) gene mutations occur in most elderly patients with memory loss. MTHFR is critical for production of S-adenosyl-l-methionine (SAM), the principal methyl donor. A common mutation (1364T/T) of the cystathionine-γ-lyase (*CTH*) gene affects the enzyme that converts cystathionine to cysteine in the transsulfuration pathway causing plasma elevation of total homocysteine (tHcy) or hyperhomocysteinemia—a strong and independent risk factor for cognitive loss and AD. Other causes of hyperhomocysteinemia include aging, nutritional factors, and deficiencies of B vitamins. We emphasize the importance of supplementing vitamin B_12_ (methylcobalamin), vitamin B_9_ (folic acid), vitamin B_6_ (pyridoxine), and SAM to patients in early stages of LOAD.

## 1. Introduction

Most genetic research on late-onset Alzheimer’s disease (LOAD) has focused on genome-wide association studies (GWAS) that have provided low effect size results in general, with the exception of apolipoprotein E (ApoE) [1,2]. Studies of monozygotic twins with Alzheimer’s disease (AD) showed discordance in onset and progression indicating a role for nongenetic factors in disease pathogenesis [3]. For these reasons, genetic research turned to epigenetic modifications using epigenome-wide association studies (EWAS) in the last few years [4,5]. Bonasio et al. [6] defined epigenetics as ‘‘the study of molecular signatures that provide a memory of previously experienced stimuli, without irreversible changes in the genetic information’’. Therefore, epigenetic refers to potentially heritable and nonheritable modifications in gene expression induced by environmental factors without changes in DNA base sequences [1]. These epigenetic processes include DNA methylation, histone modification and expression of long noncoding RNAs and noncoding microRNAs (miRNAs) that primarily repress target messenger RNAs (mRNAs) [7,8,9,10].

This review focuses on DNA methylation dynamics and other epigenetic changes, including the role of methylenetetrahydrofolate reductase (*MTHFR*) gene polymorphisms and its metabolic pathways particularly in aging and LOAD pathology [11]. We also review polymorphisms of the cystathionine-gamma(γ)-lyase (*CTH*) gene [12], the enzyme that converts cystathionine to cysteine in the transsulfuration pathway and is responsible for plasma elevation of total homocysteine (tHcy). The role of relevant nutritional factors including the B-vitamins folate, vitamin B_12_, and vitamin B_6_ status is summarized. Elevation of Hcy is important in oxidative stress contributing to the decrease of S-adenosyl-l-methionine (SAM) levels, which induce demethylation of DNA resulting in overexpression of genes involved in AD pathology such as presenilin (*PSEN1*) and beta-secretase (*BACE1*), the β-site amyloid precursor protein (APP)-cleaving enzyme that increases hypomethylation and Aβ_1-42_ deposition [9]. Moreover, epigenetic markers have also been demonstrated to be critical regulatory factors of brain function [9], not only in AD but also in other neurodegenerative diseases [1,2] as well as in aging [9]. Experimental antiaging epigenetic interventions attempt to reverse age-related changes in DNA methylation [10].

## 2. DNA Methylation Studies

### 2.1. 5-Cytosine Methylation and DNA Methyltransferases 

Methylation at the 5-position of the cytosine base (5mC) is considered a critical phase of epigenetic regulation in pathways related with neuronal development. Methylation and demethylation of cytosine-phosphate-guanine (CpG) islands is associated with alterations in local chromatin producing a long-term regulation of transcription tagging genome into active and inactive territories introducing a “masking” function [13]. Decreased levels of 5mC [1] and targeted mutations of DNA methyltransferases introduced into the germline produce severe developmental restriction [13] and finally a lethal phenotype in mice [14]. Cytosine base methylation occurs mainly at CpG dinucleotides [1]. Gene regulation is achieved by 5mC silencing gene expression via high-density CpG areas, known as CpG islands, which remain largely unmethylated [13]. In humans, genomic DNA methylation of cytosine results from the addition of a methyl group from SAM to the cytosine, catalyzed by DNA methyltransferases (DNMT1, DNMT3A, and DNMT3B) [9]. In addition to 5mC, hydroxymethylation at the 5-position of the cytosine base (5hmC) derived from the oxidation of methylated cytosines by ten-eleven translocation (TET) enzymes is another epigenetic regulatory mechanism, which is particularly abundant in the brain. The TET family of enzymes catalyzes Fe (II)- and alpha-ketoglutarate (α-KG)–dependent oxidation reactions, [9] and produces the initial step of oxidation of 5mC to 5hmC. TET enzymes also participate in the conversion of 5-formylcytosine (5fC) to 5-carboxylcytosine (5caC); this cycle ends when 5caC is excised by a thymine-DNA glycosylase (TDG) [9].

In humans, DNA methyltransferases are involved in tumor transformation and progression resulting in genome-wide hypomethylation of tumor cells and silencing of tumor-suppressor genes [15]; also, *DNMT3A* mutations have been associated with poor prognosis in acute myeloid leukemia [15]. *DNMT1* mutations occur in hereditary sensory and autonomic neuropathy type 1 (HSAN1) [15]. In mice, *DNMT1* mutations induce global hypomethylation along with cortical and hippocampal neuronal dysfunction causing neurodegeneration with severe deficits in learning, memory and behavior [16]. Hypomethylated excitatory neurons have postnatal maturation defects including abnormal dendritic arborization and impaired neuronal excitability [16]. Grossi et al. [17] used artificial neural network analysis to illustrate how low cobalamin; low folate and high Hcy are linked to AD. Low *PSEN1* methylation was linked to low folate levels and low promoter methylation of *BACE1* and *DNMT* genes. High levels of folate-vitamin B_12_ and low Hcy promoted methylation of genes required for DNA methylation reactions (*DNMT1*, *DNMT3A*, *DNMT3B*, and *MTHFR*) [18].

### 2.2. DNA Methylation in Alzheimer’s Disease

Early studies of DNA methylation in LOAD from peripheral blood lymphocytes [19,20], brain biopsies and autopsy material [21,22,23,24,25,26,27,28,29] demonstrated variable results of cytosine methylation at CpG dinucleotides. Wang and coworkers [30] studied postmortem prefrontal cortex tissue and peripheral lymphocytes of AD patients and showed that specific loci in *MTHFR* gene promoter regions were hypermethylated compared to healthy controls. Ellison and collaborators [31] using gas chromatography/mass spectrometry found abnormal levels of 5mC and 5hmC in the superior and middle temporal gyri, hippocampus and parahippocampal gyrus in early stages of AD, as well as in frontotemporal lobe degeneration and Lewy body dementia; these global values returned to control levels as the disease progressed suggesting that methylation changes occur in early stages of neurodegenerative dementias. Chouliaras et al. [32] confirmed the presence of significant decreases in levels of 5mC and 5hmC in the hippocampus of AD patients compared with negative controls. Levels of 5mC were inversely proportional to the deposition of neurofibrillary tangles in the same hippocampal cells. Hernández et al. [33] studied DNA methylation patterns of cortical pyramidal layers in 32 brains of patients with LOAD demonstrating hypermethylation of synaptic genes and genes related to oxidative stress including *HOXA3*, *GSTP1*, *CXXC1-3* and *BIN1*.

One of the major problems of initial methylation studies was the small sample size. This was solved by De Jager and collaborators [4] utilizing one of the largest clinicopathological studies to date, the Religious Orders Study, with 708 brains to assess the methylation state of the brain’s DNA correlated with AD pathology. Almost a half-million CpGs were interrogated including CpGs in the *ABCA7* and *BIN1* regions. The authors also identified genes whose RNA expression was altered in AD including *ANK1*, *CDH23*, *DIP2A*, *RHBDF2*, *RPL13*, *SERPINF1* and *SERPINF2. ANKYRIN* 1 (*ANK1*) and *RHOMBOID5* (*RHBDF2*) genes are involved in the protein tyrosine kinase 2-beta (*PTK2B*) gene network, a LOAD gene that is a key element of the calcium-induced signaling cascade involved in modulating the activation of microglia and macrophages, as well as in the transport of TNFα converting enzyme (ADAM17) from the cell surface. 

Absence of *RHBDF2* in mice impacts the normal release of TNFα [4] activated astrocytes in the vicinity of neuritic plaques that overexpress *CADHERIN23* (*CDH23*) gene. DIP2A functions as a cell surface protein and connects directly to the known *SORTILIN RELATED RECEPTOR 1* (*SORL1*) susceptibility gene that is involved in the APP susceptibility network and amyloid processing [4]. Both *SERPIN PEPTIDASE INHIBITORS* (*SERPINF1* and *SERPINF2*) interact with elements of amyloid processing. *SERPINF1* mRNA expression is reduced in LOAD and when knocked-out in vitro leads to reduced neurite outgrowth [4].

A Religious Orders companion study by Lunnon and coworkers [5] found robust association between differences in methylation, mRNA levels, and Braak & Braak staging. The severity of Alzheimer’s disease is defined in neuropathology by the presence of tau-based neurofibrillary tangles ranging from early stages (I and II) to extensive neocortical involvement in Braak & Braak stages V and VI in advanced disease. Dysregulation of DNA methylation occurred earlier in brain areas affected at onset by AD and appeared to have stronger effects (28.7%) than the combination of ApoE and other risk genes (13.9%) identified by GWAS [1,2], indicating the importance of epigenetic changes in AD. Additional studies by Yu et al. [34] confirmed the association of DNA methylation in *SORL1*, *ABCA7*, *HLA-DRB5*, *SLC24A4*, and *BIN1* genes with pathological diagnosis of AD including both Aβ load and tau tangle density. RNA expression of transcripts of *SORL1* and *ABCA7* was associated with tau tangle density, and the expression of *BIN1* was associated with Aβ load [34]. Moreover, Lunnon et al. [5] found hypermethylation of the *ANK1* gene in the entorhinal cortex, superior temporal gyrus and prefrontal cortex in LOAD. These findings confirm that AD involves significant disruption of DNA methylation. Epigenetic age-associated alterations of DNA methylation have also been reported in animal models of AD, in particular global DNA hypomethylation in the J20 model and DNA hypermethylation in the triple transgenic 3xTg-AD model [35].

## 3. miRNAs Epigenetic Effects

Long noncoding RNAs and noncoding microRNAs (miRNAs) that primarily repress target messenger RNAs (mRNAs) play a pivotal role in oncology, cardiovascular diseases and dementia [7,8]. In AD, the miRNA-125b is overexpressed enhancing neuronal apoptosis and tau phosphorylation by activation of cyclin-dependent kinase 5 (CDK5) and p35/25. *FORKHEAD BOX Q1* (*FOXQ1*) is the direct target gene of miR-125b [7]. Patrick and coworkers [8] studied the role of miRNA-132, miRNA-129 and miRNA-99 in the dorsolateral prefrontal cortex of more than 500 brain samples demonstrating a small number of specific alterations on target genes such as *EP300* that encodes p300, a histone acetyltransferase that regulates transcription in the cortex of subjects with AD.

## 4. Transsulfuration Metabolic Pathways and Remethylation Defects

**Figure 1:**
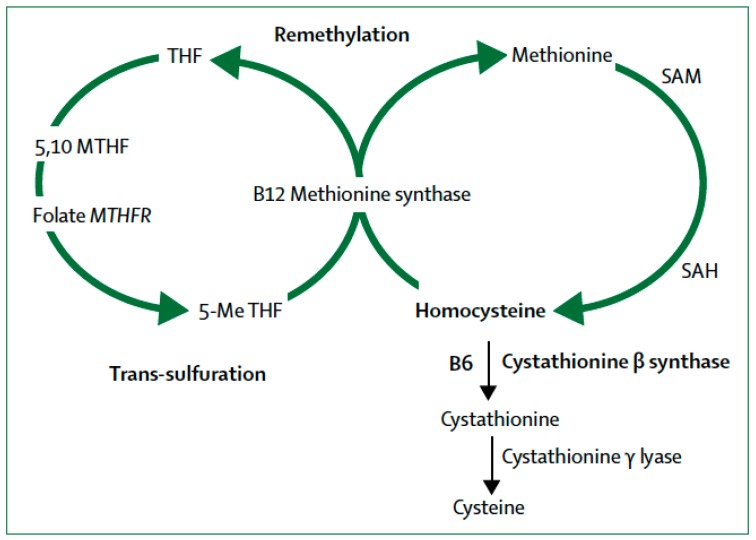
Homocysteine metabolism: B12 = cobalamin (vitamin B12). B6 = pyridoxine (vitamin B6). MTH = methylenetetrahydrofolate. MTHFR = methylenetetrahydrofolate reductase. SAM = S-adenosyl- methionine. SAH = S-adenosylhomocysteine. 5-Me THF = 5-methyl tetrahydrofolate. (From [53]).

The metabolism of sulfur-containing amino acids in the transsulfuration pathway involves the transfer of the sulfur atom of methionine to serine to produce cysteine (Figure 1). Methionine first reacts with ATP to form S-adenosyl-l-methionine (SAM), then S-adenosyl-homocysteine (SAH) and finally, homocysteine. Plasma elevation of total homocysteine (tHcy) or hyperhomocysteinemia may result from congenital deficiency of cystathionine β-synthase (CBS) leading to homocystinuria, or more frequently from polymorphisms of the cystathionine γ lyase (*CTH*) gene (OMIM *607657; EC 4.4.1.1.) in chromosome 1 (1p31.1) [36]. CTH is the enzyme that converts cystathionine to cysteine, the last step in the transsulfuration pathway. Wang et al [12] demonstrated that a single nucleotide polymorphism (SNP), namely c.1364G > T in exon 12 of the *CTH* gene causes elevation of tHcy and cystathioninuria. Caucasian subjects homozygous for the *CTH* 1364T/T SNP showed elevation of tHcy that reached effects sizes similar to those caused by the 677C > T *MTHFR* gene polymorphism [12].

Closely related to the transsulfuration pathway are the remethylation defects resulting from the failure to convert homocysteine to the amino acid methionine (Figure 1). This pathway requires the integrity of the gene encoding methylenetetrahydrofolate reductase (*MTHFR*) required for the interaction of folate and cobalamin (vitamin B_12_). Folate provides the methyl group required for the remethylation pathway (Figure 1) to finally produce SAM, the main methyl donor for epigenetic processes. The human *MTHFR* gene (OMIM *607093; EC 1.5.1.20) is localized in chromosome 1 (1p36.3) and it encodes for 5,10-methylenetetrahydrofolate reductase (MTHFR) [37]. This enzyme catalyzes the conversion of 5,10-methylenetetrahydrofolate to 5-methyltetrahydrofolate, a co-substrate with vitamin B_12_ for the remethylation of homocysteine to methionine [11]. Mutations of this gene occur in 10–15% of the population and the resulting MTHFR deficiency affects the production of methionine and SAM. Patients with AD have low levels of SAM in the CSF [38].

*MTHFR* gene polymorphisms cause enzyme thermolability and involve C-to-T substitution at nucleotide 667 and A-to-C at nucleotide 1298; these *MTHFR* mutations have been associated with homocystinuria, neural tube defects, preeclampsia, cleft lip and cleft palate, cerebrovascular disease, and psychiatric disorders including susceptibility to depression and schizophrenia [39,40]. Population-based international studies showed no increased risk of dementia in subjects with *MTHFR* polymorphisms [41,42]. In Japan, Nishiyama et al. [43] found a slight association of the *MTHFR*-C667T polymorphism with senile cognitive decline in men but not with AD. In Australia, a causal link between high tHcy and incident dementia was demonstrated [44] but the study lacked power to determine an effect of the *MTHFR*-C667T genotype. In contrast, de Lau and collaborators [45] in the normal elderly population of the Rotterdam Study observed that the *MTHFR*-C665T genotype was associated with elevated tHcy but not with cognitive loss or white matter lesions. In a small patient population in Tunisia [46], the *MTHFR*-A1298C mutation was associated with susceptibility to AD. As mentioned earlier, Román [11] found a very high frequency (above 90%) of *MTHFR* gene mutations in an elderly population attending a memory clinic in the USA, with diagnoses ranging from mild cognitive impairment (MCI) to LOAD; about 65% had single mutations; the *MTHFR*-C667T mutation was found in 58.5% of the patients and 41.5% had the *MTHFR*-A1298C mutation whereas 20% were compound heterozygous for both mutations [11].

### MTHFR and Epigenetic Drift

In 2005, a multinational study of identical twins by Fraga and collaborators [47] first demonstrated that whereas DNA methylation and histone acetylation in young identical twins are indistinguishable, older identical twins showed substantial differences; epigenetic changes were up to four times greater than those of young twin pairs. The authors concluded that this “epigenetic drift” was associated with aging [47]. Epigenetic drift of identical twins with aging also occurs among a large number of animal species [48] following a non-Mendelian pattern. In identical twins with AD, the prognosis and onset of AD can differ by more than ten years [3,49,50]; young identical twin pairs are essentially indistinguishable in their epigenetic markings while older identical twin pairs show substantial variations. Breitner et al. [50] suggested that twins with a history of systemic infection developed AD at an earlier onset than their identical twin. Epigenetic drift can be caused by lifestyle, diet, infections, folate status, homocysteine status or toxic exposure [51]. Wang et al. [30] demonstrated that the *MTHFR* gene promoter in the brain displayed high interindividual variance in DNA methylation among twins. The methylation level of *MTHFR* and *APOE* in individuals 30 years of age apart decreased by 10.6%, whereas in patients with AD the methylation level increased by 6.8%. The epigenetic drift increases with age particularly in genes that play pivotal roles in removing β-amyloid such as *APOE* and among methylation genes such as *MTHFR* and *DNMT1* [9,52].

## 5. Homocysteine (Hcy): A Risk Factor for Cognitive Loss and Dementia

Hcy is a sulfur-containing amino acid produced in the transsulfuration pathway (Figure 1) from the reaction of methionine with ATP to form SAM, then SAH and finally homocysteine. Homocystinuria due to congenital deficiency of the *CBS* gene causes hyperhomocysteinemia. Polymorphisms of the *CTH* and *MTHFR* genes are common genetic causes of hyperhomocysteinemia [38,39]. The remethylation pathway (Figure 1) involves reactions enzymatically mediated by MTHFR requiring as co-substrates the B-group vitamins folic acid (vitamin B_9_) and cobalamin (vitamin B_12_) for the remethylation of homocysteine to methionine. Pyridoxine (vitamin B_6_) is required by CBS for the conversion of homocysteine to cysteine (Figure 1).

### 5.1. Hyperhomocysteinemia is an Independent Vascular Risk Factor

Elevation of plasma or serum tHcy (hyperhomocysteinemia) is an independent vascular risk factor linked to coronary disease, peripheral vascular disease, stroke and small-vessel cerebrovascular disease [53]. More importantly, elevated tHcy is considered a risk factor for dementia and cognitive decline in the elderly, particularly in association with low levels of folate and cobalamin [54,55]. A number of studies in cognitively normal elderly subjects, demonstrated that baseline tHcy is a strong and independent predictor of cognitive decline after observation periods ranging from 3 years (USA, *n* = 321 men [55] and Sydney, Australia, *n* = 889 [56]), 4 years (France, *n* = 1241) [57], 5 years (Wales, UK, *n* = 32) [58], 6 years (Norway, *n* = 2189) [59], 7 years (Finland *n* = 274) [60] and up to 10 years (UK, *n* = 691) [61]. In the Finland cohort [60], the magnetic resonance imaging (MRI) study demonstrated the association of higher baseline vitamin B_12_ and holotranscobalamin levels with a decreased rate of total brain volume loss during 8 years of the study period [62]. Increased tHcy levels were associated with faster rates of total brain volume loss and with progression of white matter hyperintensities among participants with hypertension (systolic blood pressure > 140 mm Hg) [62].

Regarding the risk of AD associated to elevated tHcy, in the Framingham Study, Seshadri and colleagues [63] demonstrated in elderly subjects (mean age, 76 years) that raised tHcy above 14 μmol/L nearly doubled the risk of LOAD over a period of 8 years. Similar findings were corroborated in two large Finnish [60,64] and Australian [65] cohorts. In 2008, Smith [66] performed a comprehensive review of cross-sectional and prospective studies involving >46,000 subjects and confirmed the association between elevated tHcy and cognitive deficit or dementia.

According to a recent international consensus statement [67], moderately raised homocysteine (>11 μmol/L) increases the relative risk of dementia in the elderly 1.15 to 2.5 fold, and the Population Attributable risk from 4.3 to 31% [67]. From the Public Health viewpoint, homocysteine-lowering treatment with B vitamins that markedly slows down the rate of brain atrophy and cognitive decline in the elderly offers the possibility that, in addition to folic acid fortification, mandatory methylcobalamin supplementation should also be considered for the prevention of LOAD [67].

### 5.2. Genetic and Nongenetic Causes of Hyperhomocysteinemia

Elevation of tHcy is caused by numerous factors including advancing age, diet, supplementation of B-vitamins, obstructive sleep apnea, smoking, *Helicobacter pylori* infection, and renal failure, among others [53,54]. As indicated earlier, both *CBS* gene polymorphisms and the C667T and the A1298C SNPs in the *MTHFR* gene decrease the activity of the MTHFR enzyme leading to hyperhomocysteinemia. Minagawa et al. [68] found that elevated Hcy inhibits the dimerization of ApoE3 and reduces ApoE3-mediated high-density lipoprotein (HDL) concentrations involved in degradation of soluble Aβ within microglia. ApoE4 was not affected; in patients with hyperhomocysteinemia the CSF levels of ApoE3 dimers were significantly lower than in controls. Minagawa and colleagues [68] suggested that the effects of elevated Hcy on ApoE3 contribute to the pathogenesis of AD. Smith and Refsum [54] reviewed the proposed mechanisms responsible for the harmful cognitive effects of hyperhomocysteinemia (Table 1). These include impaired endothelial function with reduced inducible nitric oxide synthase; augmented oxidative stress and decreased activity of key antioxidant enzymes; raised generation of the superoxide anion; alterations of lipid metabolism with increased cholesterol synthesis and reduced synthesis of apolipoprotein 1; and, carotid stenosis and induction of thrombosis [69,70].

Hyperhomocysteinemia induces a decrease in the SAM-dependent synthesis of catecholamines including dopamine, norepinephrine, and epinephrine, as well as non-catecholamine neurotransmitters such as melatonin and serotonin (5-HT) that contribute to development of depression [69]. Moreover, elevated tHcy produces two neurotoxic products, homocysteic acid (HCA) and cysteine sulfinic acid (CSA), which are agonists of the N-methyl-D-aspartate (NMDA) glutamate receptor, with neurotoxic effects on dopaminergic neurons derived from excessive Ca^++^ influx and reactive oxygen generation [70]. The beneficial effects of B-group vitamins on elevated tHcy will be reviewed next.

## 6. Folate Metabolism

Vitamin B_9_ or folic acid (from the Latin *folium*, leaf) is abundantly found in green leafy vegetables. Folate is vital for cell development and growth given its role in numerous biochemical one-carbon (methyl-group, –CH_3_) reactions, many of them critical for cognition. The Nun Study [71] first provided epidemiological and neuropathological data demonstrating that limited lifetime consumption of salads with low blood folate levels increased the risk of cognitive decline and dementia. Also, the severity of the atrophy in the neocortex and of the Alzheimer disease lesions were strongly correlated with low serum folate levels; none of 18 other nutrients, lipoproteins, or nutritional markers measured in the study correlated with the atrophy [71]. Further studies confirmed that normal cognitive scores were highly associated with elevated blood folate despite the neuropathological evidence of LOAD brain lesions [72].

The primary methyl-group donor for DNA methylation reactions is 5-methyl-tetrahydrofolate (CH_3_-THF) required for the transformation of homocysteine into methionine mediated by methionine synthase with cobalamin (vitamin B_12_) as a cosubstrate (Figure 1), leading to the synthesis of SAM. Also, CH_3_-THF is critical in the de novo purine synthesis to convert dUMP (deoxyuridylate) into dTMP (thymidylate) for DNA and RNA synthesis, DNA repair or replication. Several forms of cancer are associated with epigenetic differential methylation causing disturbances in nucleotide synthesis; for instance, hypermethylation may inhibit tumor suppressors. Folate, therefore, is a B-vitamin that plays an important role as a precursor in the epigenetic regulation of gene expression, DNA stability, DNA integrity and mutagenesis. Abnormal folate status has been associated with neural tube defects, cardiovascular and cerebrovascular diseases, cleft lip and palate, neurodegenerative diseases, schizophrenia and depression [40,73,74].

### Telomeres and Folate Levels

Telomeres protect chromosomes from abnormal combination and degradation. The shortening of telomeres’ cap serve as a signature of cell division history, acting as biomarker of aging. In peripheral leukocytes, short telomere length is associated with increased risk of cognitive decline and LOAD [75,76]. Low folate levels are associated with short telomeres due to DNA damage in the telomeric region. Telomere length is epigenetically regulated by DNA methylation and directly influenced by folate status, a process independent of DNA damage due to uracil incorporation. Shorter telomeres occur with age, infection, stress, and chronic diseases including LOAD [75].

Paul and collaborators [76] observed that decreased plasma folate concentration to <11.6 μmol/L was correlated with a decrease in mean telomere length. In this population, homozygous carriers of the *MTHFR*-C677T gene mutation showed decreased levels of plasma folate [77]. Decreased serum folate induces anomalous integration of uracil in place of thymidine in DNA [78], a mechanism corrected by folic acid supplementation. Troesch, Weber and Mohajeri [79] summarized the importance for the development of LOAD of reduced SAM-dependent methylation reactions due to genetic factors along with reduction of folate, vitamin B_6_ and vitamin B_12_ levels. The resulting elevation of Hcy levels and the reduced capacity to synthetize, methylate and repair DNA, along with the impaired modulation of neurotransmission, appears to favor the development of AD particularly when combined with increased oxidative stress, particularly in ApoE ε4 carriers [80].

## 7. Vitamin B_12_ Deficiency and β-amyloid Deposition

Smith, Warren and Refsum [81] have recently provided a comprehensive review of vitamin B_12_. Only bacteria can biosynthesize vitamin B_12_; in humans B_12_ from the diet is a cofactor for the enzymes methionine synthase and l-methyl-malonyl-CoA mutase. B_12_ deficiency results in build-up of homocysteine and lack of interaction with folate that is trapped as CH_3_-THF leading to depletion of tetrahydrofolates used in thymidylate and purine synthesis blocking DNA for the production of red cells in the bone marrow. B_12_ deficiency impedes cellular proliferation and protein synthesis and thereby causes development of megaloblastic anemia [81].

### 7.1. Clinical Manifestations of Vitamin B_12_ Deficiency

In 1920, pernicious anemia—a fatal form of a megaloblastic anemia—was successfully treated by adding liver to the diet. In 1955, Dorothy Hodgkin used crystallography to first identify the molecular structure of cyanocobalamin or vitamin B_12_ from the deep-red cyanide-containing pigment isolated from liver tissue. Pernicious anemia was the first disease to be identified as caused by vitamin B_12_ deficiency [81]. 

Stabler [82] reviewed the clinical manifestations of vitamin B_12_ deficiency. In addition to megaloblastic anemia, acidemia from elevation of serum methylmalonic acid (MMA), and methylmalonic aciduria, the neurological manifestations of pernicious anemia include memory loss and cognitive decline, visual disturbances from optic nerve neuropathy, burning and painful sensations in hands and feet from peripheral neuropathy, and spinal cord involvement with subacute combined degeneration resulting in loss of proprioception from dorsal column involvement and pyramidal tract symptoms such as paralysis and incontinence.

### 7.2. Measuring Total Serum B_12_ Levels

Dietary sources of B_12_ include liver, meat, fish, shellfish and dairy products; vegans are prone to B_12_ deficiency [81,82]. Vitamin B_12_ deficiency occurs from inborn metabolic errors, alterations of B_12_-binding proteins including *haptocorrin* (HC) found in saliva, *intrinsic factor* (IF) produced by parietal cells in the stomach (pernicious anemia is associated with anti-parietal-cell and anti-IF auto-antibodies), and *transcobalamin* (TC), which binds B_12_ to facilitate uptake by the cells [81]. According to Stabler [82], measurement of total serum B_12_ levels is unsatisfactory because it reflects B_12_ that is bound to either HC or TC, and up to 60% of bound materials are cobalamin analogues (corrinoids). Therefore, “normal” total serum B_12_ levels can mask deficiency if serum contains relatively large amounts of cobalamin analogues [83]. Levels below 200 pg/mL usually indicate biochemical B_12_ insufficiency. Serum B_12_ < 350 pg/mL along with tHcy > 14 µmol/L indicate metabolic B_12_ deficiency [81,82]. For this reason, holotranscobalamin, MMA and tHcy levels should be included in the evaluation of a patient suspected of having B_12_ deficiency [83].

### 7.3. Causes of Vitamin B_12_ Deficiency

Other than pernicious anemia resulting from presence of anti-parietal-cell and anti-IF autoantibodies, other causes of B_12_ deficiency include atrophic body gastritis, *Helicobacter pylori* infection, malabsorption of vitamin B_12_, gastrectomy, gastric bypass or other bariatric surgery, inflammatory bowel disease, tropical sprue, use of metformin, anticonvulsants, proton-pump inhibitors and other drugs to block stomach acid, and vegetarian diets low in meat and dairy products. Hemodialysis patients, nitrous oxide inhalation, and cholinesterase inhibitors in LOAD patients [84] also increase the risk of vitamin B_12_ deficiency.

Epidemiological studies have shown that prevalence of vitamin B_12_ deficiency increases with age [85,86], due to decreased saliva (e.g., dry eyes-dry mouth of Sjögren syndrome) [87] and gastric atrophy with deficits respectively of haptocorrin and intrinsic factor. Andrès and colleagues [88] have emphasized that as many as 20% of elderly people may have unrecognized B_12_ deficiency due to food-cobalamin malabsorption plus insufficient dietary intake. According to Spence [89], metabolic B_12_ deficiency occurs in 30% of vascular patients older than 71 years, increasing to as many as 40% in patients above age 80 years; these patients usually have plasma levels of tHcy >14 µmol/L resulting from B_12_ deficiency. Inadequate supply of B_12_ and folic acid is not only a strong and independent vascular risk factor particularly for subcortical ischemic small-vessel disease [90], a common and important contributor to cognitive impairment and memory complaints in the elderly, but also enhancing the development of LOAD [91]. Animal experimental data confirms the importance of B-vitamin deprivation in the expression of AD [92].

### 7.4. Effects of B-Group Vitamins on Cognition: Negative Clinical Trials

An international consensus [67] provided a comprehensive explanation of the negative results of meta-analyses [93] based on reviews of the results from a number of inadequately controlled clinical trials; most participants in those trials were enrolled in post-hoc studies which were not designed primarily to assess cognition. Usually, these were short-duration trials without baseline cognitive assessment and results were based on post-hoc brief cognitive assessments; only a few of these studies assessed the incidence of dementia or mild cognitive impairment. 

In contrast, solid positive results were obtained in the Oxford Project to Investigate Memory and Ageing (OPTIMA) trial [94,95,96] that used comprehensive neuropsychological evaluations plus brain imaging end-points. The results of this trial indicate that supplementation of B_12_, pyridoxine, and folic acid in subjects with MCI and hyperhomocysteinemia decreases tHcy resulting in improved episodic memory and global cognition [95], and most importantly, brain imaging demonstration of slowing of the progression of the brain atrophy in areas affected by AD [96]. Current recommendation is to provide oral supplementation of methylcobalamin 1000 µg/d, folic acid 800 µg/d and pyridoxine 100 mg/d.

## 8. SAM in Depression and Cognitive Loss

As described above (Figure 1) SAM is the main methyl-group donor for the methylation reactions reviewed here; as well as for synthesis of neurotransmitters, proteins, nucleic acids, phospholipids, and myelin. SAM has been used as an adjuvant for the treatment of depression [97]. Linnebank et al. [38] demonstrated a decrease of SAM in the cerebrospinal fluid (CSF) of patients with LOAD, affecting mainly ApoE ε4 carriers. According to Dayon et al. [98], plasma levels of one-carbon metabolites predicted cognitive decline. Despite the enhancing effects of SAM on antidepressants, no conclusive clinical trials of SAM have been reported [99].

## 9. Conclusions

It is established that the damaging effects of deficiencies of folate and cobalamin and the resulting elevation of tHcy contribute to the development of LOAD [67]. The numerous detrimental effects of elevated tHcy include, among others, endothelial and cerebrovascular damage of large-vessels as well as small-vessel disease [90]; activation of tau kinases; inhibition of methylation reactions; epigenetic effects on the β-amyloid pathway; reduced protein phosphatase-2A; and, impaired formation of phosphatidylcholine. Adequate supply of B-vitamins in the elderly, particularly in subjects with *MTHFR* and *CTH* gene mutations, appears to be critical to prevent the development of cognitive decline and to halt the progression of LOAD.

## Figures and Tables

**Table 1:** Harmful effects of homocysteine on vascular function and cognition (modified from Smith & Refsum [54]).

Proposed Mechanisms	
Vascular Mechanisms	
1	Impairs endothelial function reducing inducible NO synthase
2	NO-mediated endothelial dysfunction in brain vasculature
3	Causes a leaky blood-brain barrier
4	Induces thrombosis
5	Cerebrovascular ischemia leading to neuronal death and tau tangle deposition
6	Affects lipid metabolism increasing cholesterol synthesis
7	Reduces synthesis of apolipoprotein 1
8	Causes cerebral amyloid angiopathy
**Neuronal Mechanisms**	
1	Direct activation of NMDA receptor causes excitotoxic neuronal death
2	Homocysteic acid and cysteine sulfinic acid activate NMDA receptor causing neuronal death by excitotoxicity
3	Oxidative stress induced by generating superoxide and reactive oxygen species
4	Decreased activity of antioxidant enzymes
5	Formation and deposition of β-amyloid
6	Potentiates neurotoxic effects of β-amyloid by itself or via homocysteic acid
7	Activates tau kinases, such as Cdk5, causing tau tangle deposition
8	Triggers the cell cycle in neurons, leading to tangle formation and cell death
9	Causes DNA damage, limits DNA repair, leading to apoptosis
10	Increases SAH inhibiting methylation reactions, such as DNA cytosine methylation in promoters for amyloid genes, causing epigenetic effects
11	Inhibits PP2A activity leading to tau tangle deposition
12	Inhibits methylation of phosphatidyletanolamine
13	Stimulates endoplasmic reticulum stress response leading to amyloid formation
14	Activates the immune system
15	Decreases SAM-dependent synthesis of catecholamines and other neurotransmitters
